# Association of intramural fat deposition in the interatrial septum with focal atrial tachyarrhythmias originating near the atrioventricular node

**DOI:** 10.1007/s00380-013-0447-6

**Published:** 2013-12-06

**Authors:** Shingo Maeda, Takeshi Sasaki, Yasuhiro Shirai, Kensuke Ihara, Mihoko Kawabata, Yasuhiro Yokoyama, Saman Nazarian, Stefan L. Zimmerman, Kenzo Hirao

**Affiliations:** 1 Heart Rhythm Center, Tokyo Medical and Dental University, 1-5-45 Yushima, Bunkyo-ku, Tokyo, 113-8519 Japan; 2 Department of Cardiology, Johns Hopkins University, Baltimore, MD USA; 3 Department of Radiology and Radiological Science, Johns Hopkins University School of Medicine, Baltimore, MD USA

**Keywords:** Atrial tachyarrhythmia, Fat, Atrioventricular node

## Abstract

We describe a case with three focal atrial tachycardias (ATs) and focal atrial fibrillation (AF) originating from the interatrial septum (IAS) near the atrioventricular node (AVN). Contrast-enhanced computed tomography demonstrated the association of fat deposition within the anterior IAS near the AVN with successful ablation sites of these ATs and AF. This is the first report that the intramural fat deposition in the IAS could be associated with the formation of AT and AF re-entry circuits originating near the AVN.

## Introduction

Atrial tachycardia (AT) originating near the atrioventricular node (AVN) has been shown to originate from calcium channel-dependent tissue located close to the AVN [1]. However, its pathogenesis remains unclear. Further study has concluded that increased pericardial fat is associated with a higher risk of atrial fibrillation (AF) [2–4] and coronary artery disease [5]. The relationship between fat depositions within the interatrial septum (IAS) and atrial arrhythmias is unknown.

## Case report

**Fig. 1:**
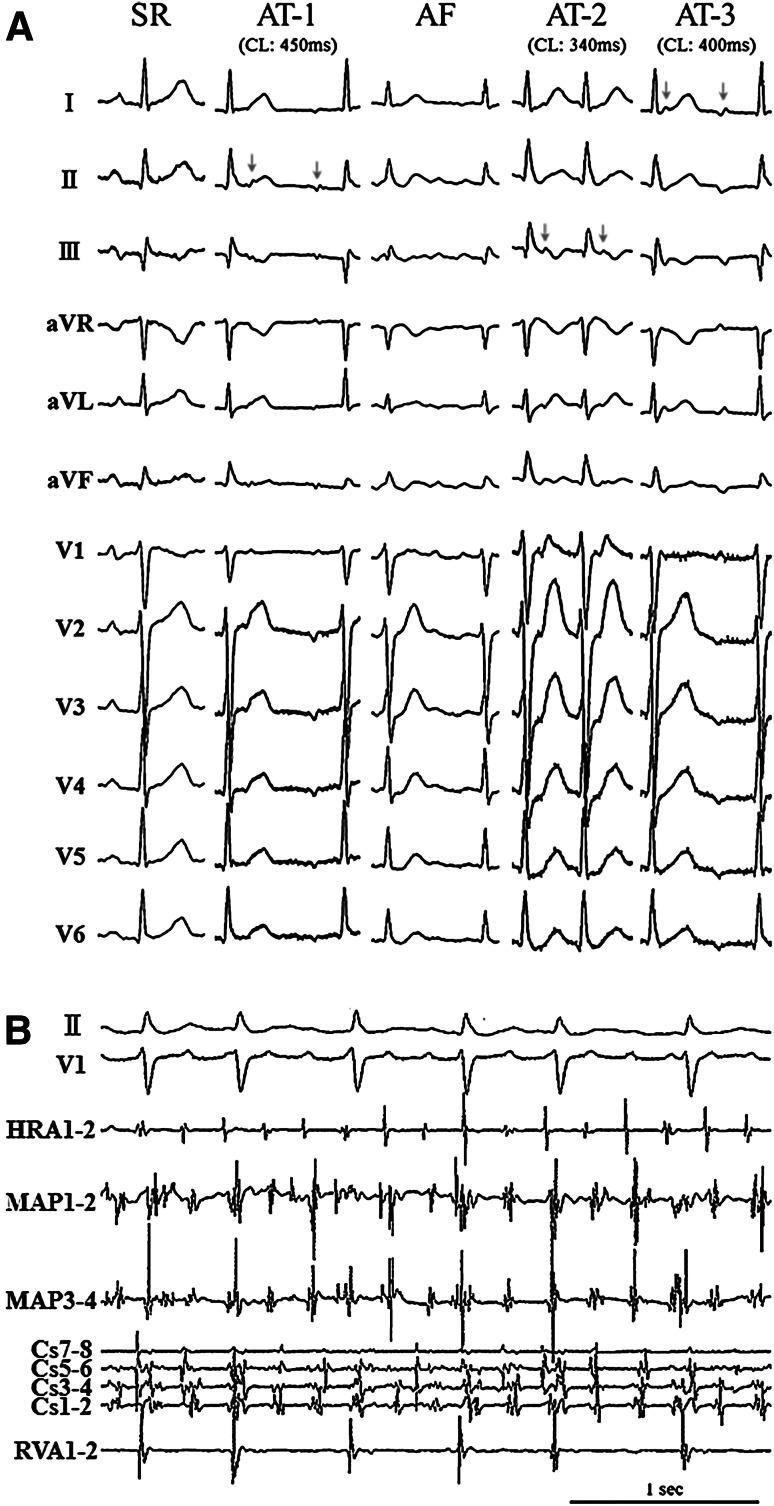
**a** Twelve-lead ECG during sinus rhythm (SR), atrial tachycardia (AT-1, AT-2, AT-3; *arrows*) and atrial fibrillation (AF). **b** Fractionated electrocardiograms during focal atrial fibrillation originating from the interatrial septum near the region of the atrioventricular node slow pathway (MAP). *HRA* high right atrium, *MAP* mapping catheter, *Cs* coronary sinus, *RVA* right ventricular apex

**Fig. 2:**
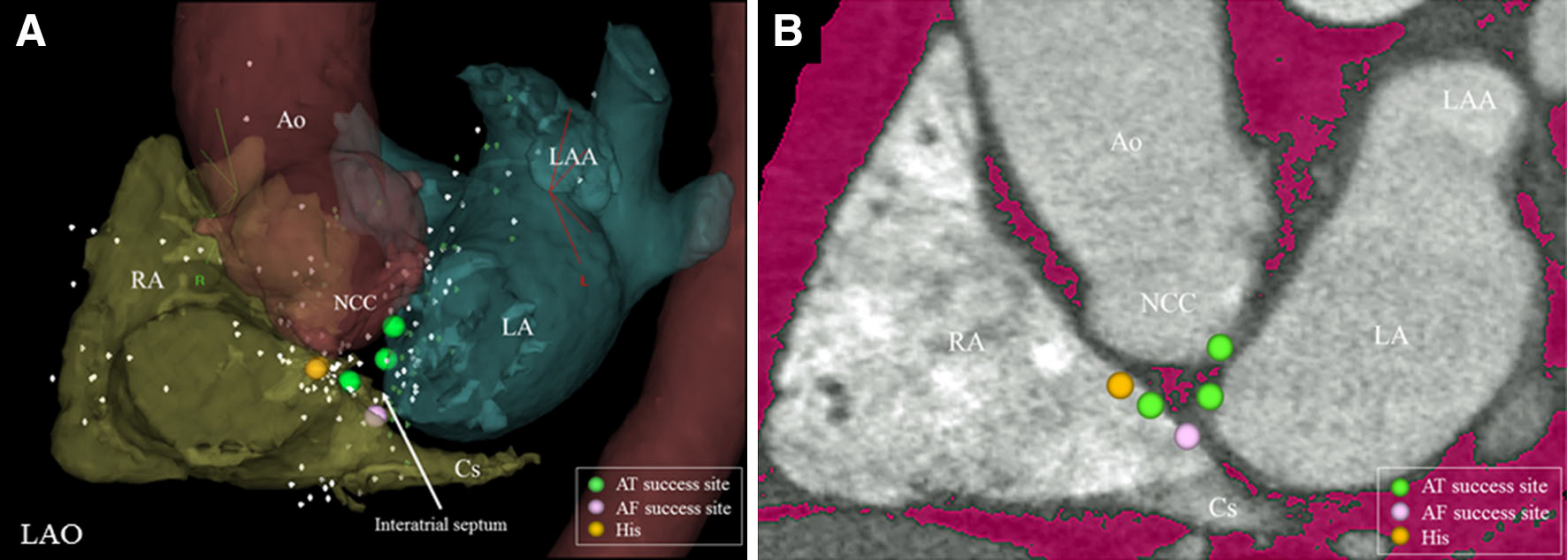
Successful ablation sites (AT: *green tag*, AF: *pink tag*) in the atrial septum near the atrioventricular node on an electroanatomic map (**a**) and the corresponding short-axis computed tomography image (ZioM900; Ziosoft, Tokyo, Japan) (**b**). *Color maps* were overlaid on CT images to mark fat-density pixels with *red color* (fat defined based on Hounsfield units between −180 and 0). Intramural fat deposition is clearly demonstrated in the interatrial septum (*red color*). *RA* right atrium, *LA* left atrium, *LAA* left atrial appendage, *Ao* aorta, *NCC* noncoronary aortic cusp, *Cs* coronary sinus, *LAO* left anterior oblique

A 69-year-old man was referred to our hospital with palpitations. His electrocardiogram showed a regular AT with narrow QRS (AT-1, Fig. 1a). Electroanatomic mapping during AT-1 demonstrated focal activation from the right atrial (RA) septum. AT-1 was eliminated by radiofrequency (RF) ablation; however, AF initiation was noted after elimination of AT-1 (Fig. 1a). Interestingly, the earliest atrial activation with a fractionated potential during AF (Fig. 1b) was observed in the RA septum posterior to the region of the AV nodal slow pathway, which was very close to the successful ablation site for AT-1. After AF terminated during focal ablation at this site, another focal AT (AT-2, Fig. 1a) originated, with a different cycle length and a different P-wave morphology. The earliest activation of AT-2 was identified in the left atrial (LA) septum just on the opposite side of the ablation site of AT-1. A third AT (AT-3) was additionally induced after AT-2 was eliminated using a transseptal approach for ablation (Fig. 1a). The earliest activation during AT-3 was observed in the noncoronary aortic cusp (NCC). AT-3 immediately terminated after RF ablation at the NCC. No further atrial tachyarrhythmia was inducible at the end of these procedures. Electroanatomic mapping demonstrated the origins of these focal atrial tachyarrhythmias, which were located in the IAS close to the AVN (Fig. 2a). Contrast-enhanced computed tomography (CT) demonstrated the downward shift of the NCC and fat deposition within the anterior IAS close to the AVN (Fig. 2b). Registration of CT images with electroanatomic map data revealed that the successful ablation sites were located across the IAS and near the His bundle.

## Discussion

This case presented both focal ATs and focal AF originating from the IAS near the AVN. Contrast-enhanced CT demonstrated a heterogeneous fat pad in the interatrial septum surrounded by focal triggers for these atrial arrhythmias. The induction of these ATs with programmed stimulation and behavior during entrainment mapping was consistent with micro re-entry as the tachycardia mechanism. These ATs may have shared a central common pathway, as suggested by the proximity of their successful ablation sites across the IAS near the AVN (Fig. 2). In addition, the mechanism of the focal AF might have been associated with the RF applications in the RA-IAS for AT-1. The local electrogram at the earliest atrial activation site during the AF presented discontinuous fractionations, as shown in Fig. 1b. This might suggest the presence of rapid localized re-entry within the IAS close to the area with fat deposition, which might have acted as a driver of AF. Previous reports have demonstrated that pericardial fat produces a number of inflammatory mediators. Given its direct apposition to the atrial myocardium, pericardial fat itself might play a critical role in the pathogenesis of AF, mediated by an increased expression of inflammatory biomarkers [6, 7]. However, the impact of intramural fat deposition in the IAS and on the pathogenesis of AT or AF has not been investigated. In this case, the origins of the induced ATs turned out to be adjacent to the areas with fat deposition in the anterior IAS near the AVN. This close association raises the question of whether intramural fat deposition in the IAS could be associated with the formation of AT and AF re-entry circuits originating near the AVN. The downward shift of the ascending aorta due to hypertension might also cause changes in atrial myocardial tissue in the IAS.

This is the first study to report that the intramural fat deposition in the IAS could be associated with the formation of AT and AF re-entry circuits originating near the AVN. Further study will be required to investigate the association of fat depositions in the IAS with atrial tachyarrhythmias originating near the AVN.

## References

[1] Yamabe H, Tanaka Y, Morihisa K, Uemura T, Enomoto K, Kawano H, Ogawa H (2010). Analysis of the anatomical tachycardia circuit in verapamil-sensitive atrial tachycardia originating from the vicinity of the atrioventricular node. Circ Arrhythm Electrophysiol.

[2] Thanassoulis G, Massaro JM, O’Donnell CJ, Hoffmann U, Levy D, Ellinor PT, Wang TJ, Schnabel RB, Vasan RS, Fox CS, Benjamin EJ (2010). Pericardial fat is associated with prevalent atrial fibrillation: the Framingham Heart Study. Circ Arrhythm Electrophysiol.

[3] Maeda S, Iesaka Y, Uno K, Otomo K, Nagata Y, Suzuki K, Hachiya H, Goya M, Takahashi A, Fujiwara H, Hiraoka M, Isobe M (2012). Complex anatomy surrounding the left atrial posterior wall: analysis with 3D computed tomography. Heart Vessels.

[4] Suenari K, Nakano Y, Hirai Y, Ogi H, Oda N, Makita Y, Ueda S, Kajihara K, Tokuyama T, Motoda C, Fujiwara M, Chayama K, Kihara Y (2013). Left atrial thickness under the catheter ablation lines in patients with paroxysmal atrial fibrillation: insights from 64-slice multidetector computed tomography. Heart Vessels.

[5] Yamashita K, Yamamoto MH, Ebara S, Okabe T, Saito S, Hoshimoto K, Yakushiji T, Isomura N, Araki H, Obara C, Ochiai M (2013). Association between increased epicardial adipose tissue volume and coronary plaque composition. Heart Vessels.

[6] Nguyen BL, Fishbein MC, Chen LS, Chen PS, Masroor S (2009). Histopathological substrate for chronic atrial fibrillation in humans. Heart Rhythm.

[7] Bambace C, Sepe A, Zoico E, Telesca M, Olioso D, Venturi S, Rossi A, Corzato F, Faccioli S, Cominacini L, Santini F, Zamboni M (2013). Inflammatory profile in subcutaneous and epicardial adipose tissue in men with and without diabetes. Heart Vessels.

